# Lipoxygenase (LOX) in Sweet and Hot Pepper (*Capsicum annuum* L.) Fruits during Ripening and under an Enriched Nitric Oxide (NO) Gas Atmosphere

**DOI:** 10.3390/ijms232315211

**Published:** 2022-12-02

**Authors:** Salvador González-Gordo, Amanda Cañas, María A. Muñoz-Vargas, José M. Palma, Francisco J. Corpas

**Affiliations:** Group of Antioxidants, Free Radicals and Nitric Oxide in Biotechnology, Food and Agriculture, Department of Stress, Development and Signaling in Plants, Estación Experimental del Zaidín, Spanish National Research Council (CSIC), Profesor Albareda 1, E-18008 Granada, Spain

**Keywords:** capsaicin, isoenzymes, genes, lipoxygenase, nitric oxide, pepper, ripening

## Abstract

Lipoxygenases (LOXs) catalyze the insertion of molecular oxygen into polyunsaturated fatty acids (PUFA) such as linoleic and linolenic acids, being the first step in the biosynthesis of a large group of biologically active fatty acid (FA)-derived metabolites collectively named oxylipins. LOXs are involved in multiple functions such as the biosynthesis of jasmonic acid (JA) and volatile molecules related to the aroma and flavor production of plant tissues, among others. Using sweet pepper (*Capsicum annuum* L.) plants as a model, LOX activity was assayed by non-denaturing polyacrylamide gel electrophoresis (PAGE) and specific in-gel activity staining. Thus, we identified a total of seven LOX isozymes (I to VII) distributed among the main plant organs (roots, stems, leaves, and fruits). Furthermore, we studied the FA profile and the LOX isozyme pattern in pepper fruits including a sweet variety (Melchor) and three autochthonous Spanish varieties that have different pungency levels (Piquillo, Padrón, and Alegría riojana). It was observed that the number of LOX isozymes increased as the capsaicin content increased in the fruits. On the other hand, a total of eight *CaLOX* genes were identified in sweet pepper fruits, and their expression was differentially regulated during ripening and by the treatment with nitric oxide (NO) gas. Finally, a deeper analysis of the LOX IV isoenzyme activity in the presence of nitrosocysteine (CysNO, a NO donor) suggests a regulatory mechanism via *S*-nitrosation. In summary, our data indicate that the different LOX isozymes are differentially regulated by the capsaicin content, fruit ripening, and NO.

## 1. Introduction

Lipoxygenase (LOX; EC1.13.11.12) is a non-heme iron-containing dioxygenase that catalyzes the bioxygenation of polyunsaturated fatty acids (PUFA), such as linoleic and linolenic acids (C18:2 and C18:3, respectively), producing the corresponding fatty acid hydroperoxides containing a cis,cis-1,4-pentadiene unit to form conjugated hydroperoxydienoic acids. LOXs can be categorized as 9- and 13-lipoxygenases, according to the carbon position (either C9 or C13) where the oxygen is inserted in both C18:2 and C18:3. These enzymes play significant functions in plant development and stress response [1,2,3,4,5,6,7]. Fatty acids are indispensable components in plant cells [8]. Among them, the 18-carbon (C18) unsaturated fatty acids, including C18:1 (oleic acid), C18:2 (linoleic acid), and C18:3 (linolenic acid) play important biological roles as precursors of jasmonic acid (JA), a hormone involved in stress response [9,10]. They are also implicated in cutin and suberin biosynthesis, which are components of the extracellular barrier [11,12]. On the other hand, from a nutritional point of view, linolenic and linoleic acids are essential PUFAs for health because human beings are unable to synthesize them endogenously [13].

Pepper (*Capsicum annuum* L) fruit is a product of great economical relevance, being considered the second most consumed horticultural product worldwide after tomato. In addition, from a nutritional point of view, it is a fruit with nutraceutical properties due to its high content of vitamins A, C, and E, carotenoids, and flavonoids, which have antioxidant properties [14]. Furthermore, many pepper varieties cultivated throughout the world are considered hot peppers since they contain capsaicin (8-methyl-N-vanillyl-6-nonenamide) and other related phenylpropanoids, which are exclusive of this plant species and mainly synthesized in the placenta of fruits. Capsaicinoids provide the pungency trait to pepper fruits where they have some physiological functions to protect the fruit against herbivores, but these compounds seem to have biomedical applications [14,15].

In previous studies, the metabolism of reactive oxygen species (ROS) systems using a sweet pepper variety (Melchor) from California-type fruits [16,17,18,19,20,21,22,23], as well as three autochthonous Spanish varieties including Padrón, Piquillo, and Alegría riojana, which have dissimilar pungency levels, has been investigated [24]. Additionally, a preliminary approach reported the fatty acid profile and LOX activity from pepper fruits at several ripening conditions [19]. The present study aims to analyze the potential relevance of LOXs at the biochemical and gene expression levels using different pepper plant organs and varieties with special attention to sweet pepper fruit during ripening and under an enriched nitric oxide (NO) atmosphere. Very recently, it has been shown that NO delays the ripening process of sweet pepper fruit and modulates its antioxidant system [21,22,23], and parallel results have been reported in tomatoes [25]. The data indicate that the LOX isozyme pattern is differentially modulated among pepper organs, fruit variety, capsaicin content, and ripening stage. Furthermore, the activity and gene expression of the different LOXs in sweet pepper fruits were regulated by NO, and this opens new windows of research on the relevance of LOX in this non-climacteric fruit.

## 2. Results

### 2.1. LOX Isozyme Profile in Pepper Plants and Ripening Stage of Fruits

**Figure 1:**
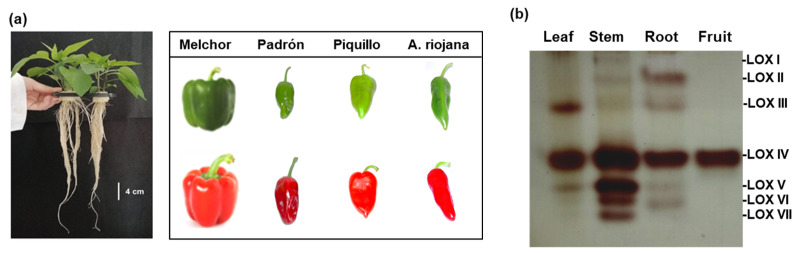
(**a**) Appearance of 30-day-old pepper (*Capsicum annuum* L. cultivar Melchor) plants and fruits (green and red) from four pepper varieties including Melchor, Padrón, Piquillo, and Alegría riojana used in this study. (**b**) In-gel lipoxygenase (LOX) activity assay in the main organs of sweet pepper fruits.

Figure 1a shows the appearance of the pepper samples (plants and fruits) used in this study, including the fruits of a California-type sweet pepper variety (Melchor) and three autochthonous Spanish varieties including Padrón, Piquillo, and Alegría riojana. The analysis of the main organ (fruit, root, stem, and leaf) of sweet pepper plants by non-denaturing PAGE allowed identifying a total of seven LOX isozymes that were designated from LOX I to LOX VII, based on their increasing electrophoretic mobility in the gels (Figure 1b). Among all of them, LOX IV was the most abundant and was present in all organs.

**Figure 2:**
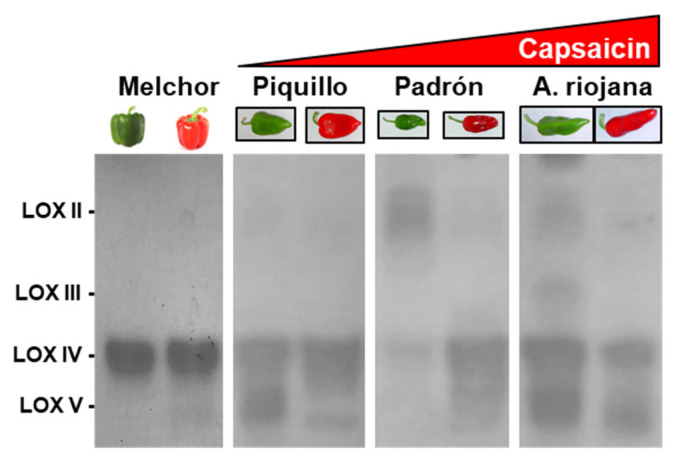
In-gel lipoxygenase (LOX) isozyme activity assay in fruits from four pepper varieties including “Melchor”, “Padrón”, “Piquillo” and “Alegría riojana”.

Considering the great diversity of pepper varieties worldwide, a comparative study was conducted of the LOX isozymes in both immature (green) and mature (red) fruits of several *C. annuum* varieties with different capsaicin content, including sweet pepper (without capsaicin) and three Spanish autochthonous pepper varieties with different pungency degrees, Padrón, Piquillo, and Alegría riojana [24]. Figure 2 shows the LOX isozyme pattern among the varieties and the ripening stage using as a reference the same nomenclature of Figure 1b. It should be noted that LOX IV was the most abundant isozyme except for green peppers from the Padrón variety, where LOX II was more prominent. On the other hand, the LOX pattern changed during ripening. In green fruits of sweet pepper (Melchor cultivar), only the LOX IV isozyme was detected. However, in ripe fruits, besides LOX IV increasing slightly, it also appeared as LOX V, which represented 13% of the total activity. In green fruits from the Padrón variety, LOX II and IV represented 79% and 21%, respectively, and in ripe fruits LOX II diminished to 13%, while LOX IV increased to 87%; moreover, LOX V appeared in ripe fruits. In the Piquillo variety, LOX II, IV, and V represented 11%, 48%, and 41%, respectively, in green fruits, while in ripe fruit, LOX II was almost maintained (9%), LOX IV moved up to 80%, and LOX V decreased to 11%. Finally, in green fruits from Alegría riojana, five LOX isozymes were detected corresponding to I, II, III, IV, and V, which accounted for 4%, 13%, 9%, 37%, and 39%, of the total activity, respectively. However, in ripe fruit, only LOX II, IV, and V were detected. LOX II slightly diminished to 9%, while LOX IV and V increased to 42% and 51%, respectively.

**Figure 3:**
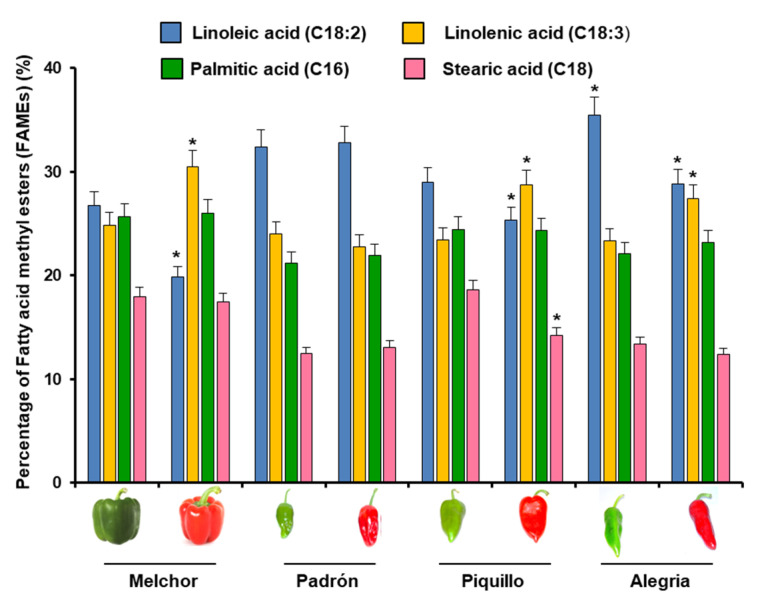
Fatty acid composition (GC/MS) of fruits from four pepper varieties with different capsaicin content expressed as percentage of fatty acid methyl esters (FAMEs) (%). Asterisks indicate that differences between values of each FA within each variety were statistically significant at *p* < 0.05, in comparison to the green stage.

LOX catalyzes the oxygenation of polyunsaturated fatty acids (PUFA). Therefore, the relative content of FAs was studied by gas chromatography/mass spectrometry (GC/MS) as a percentage of fatty acid methyl esters (FAME). The most abundant FAs in the four fruit varieties in green and ripe stages were the PUFA linoleic (C18:2) and linolenic (C18:3) acids which correspond to the omega-6 and omega-3 FA families, respectively, as well as the saturated palmitic (C16) and stearic (C18) acids (Figure 3). It was noteworthy that C18:2 was the most abundant PUFA in green fruits of all varieties, and its content decreased after the fruits ripened, except in the Padrón variety, whose content was unaffected. On the other hand, C18:3 had an inverse behavior, since their content increased in red peppers as compared to green ones, except in the Padrón variety, in which the content was unaffected. Regarding C16 and C18, it was observed that during ripening, the linoleic and stearic acid content declined in Piquillo red fruits whereas linolenic acid increased.

### 2.2. Modulation of CaLOX Genes Expression and LOX IV Activity in Sweet Pepper: Effect of Ripening and NO

**Figure 4:**
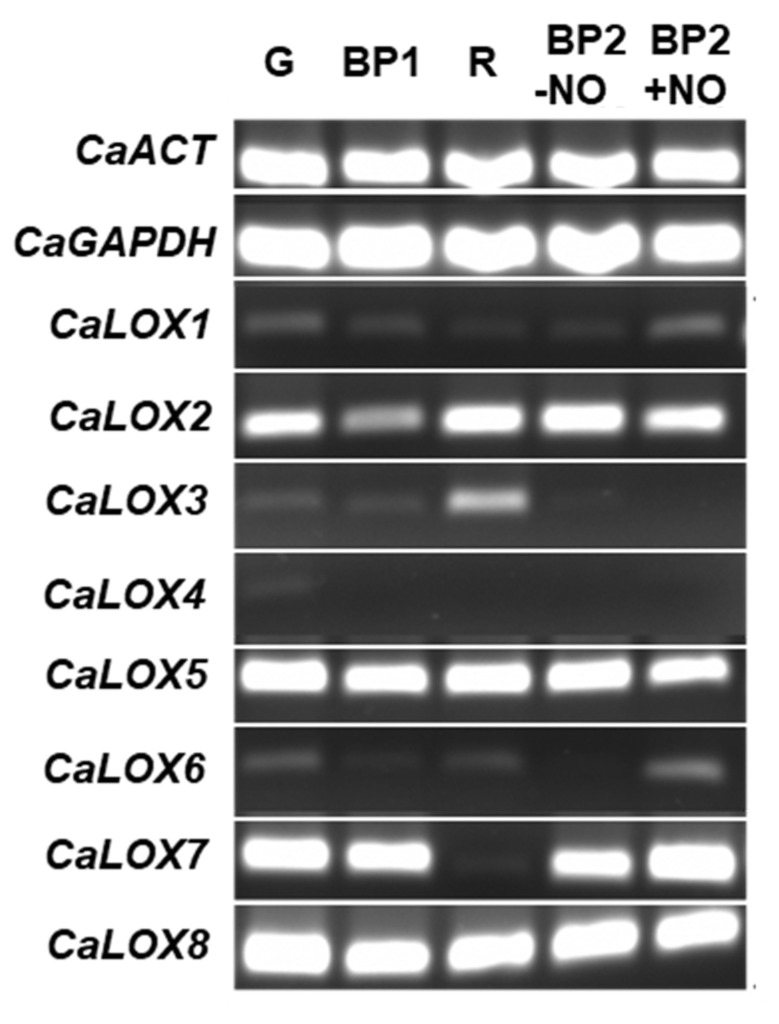
Relative expression of the *CaLOX* genes (SQ–PCR) in samples of sweet pepper fruits at different ripening stages and under NO treatment. Samples correspond to immature green (G), breaking point 1 (BP1), ripe red (R), and breaking point 2 with and without NO treatment (BP2 + NO and BP2 − NO, respectively). Supplementary Figure S1 illustrates a representative photograph of the experimental design used to analyze the pepper fruit ripening and exposed to NO treatment [23]. ACT, actin. GAPDH, glyceraldehyde-3-phosphate dehydrogenase. LOX, lipoxygenase.

Based on previous assays of sweet pepper fruits using the experimental design presented in Supplemental Figure S1, three developmental stages were established: green immature (G), breaking point (BP1), and red ripe (R). Furthermore, for the application of the exogenous NO, two additional groups were selected: fruits treated with 5 ppm NO for 1 h (BP2 + NO) and another group that was not treated with NO (BP2 -NO), which was used as control of BP2 + NO. Thus, the identification of the *LOX*-encoding genes was accomplished from the transcriptome of the sweet pepper fruit reported earlier [21]. A total of eight genes, designated *CaLOX1* to *CaLOX8*, were identified. The semi-quantitative PCR (SQ–PCR) analysis of these genes during ripening and under NO gas treatment (BP2 + NO) in comparison to untreated fruits (BP2 - NO) is shown in Figure 4. The *CaLOX* genes showed different responses during ripening and under NO enriched atmosphere (5 ppm/1 h). The expression of *LOX1* decreased 3.4-fold in ripe red (R) in comparison to immature green (G) pepper, but the NO gas exerted a positive modulation with an increase of 3.2-fold in comparison to the untreated fruit at the BP2 stage. On the contrary, *LOX2* expression increased slightly during ripening, and NO exerted a slight decrease; in both cases, the differences were not significant. *LOX3* was up-regulated during ripening about 3-fold, and at the BP2 stage, the *LOX3* was not detected. *LOX4* was only slightly detected in green pepper and was undetectable in the other conditions. *LOX5* did not seem to be affected during ripening, although NO provoked a slight down-regulation of around 1.3-fold. *LOX6* appears to be diminished about 1.9-fold after fruits ripened, although NO triggered a positive effect inducing its expression. However, *LOX7* expression drastically decreased around 32-fold in red peppers as compared to green fruits, and NO seemed to exert a positive effect of 1.4-fold. Finally, the expression of *LOX8* was slightly diminished, but not significantly, by the ripening process (G to R) or NO (BP2 + NO).

**Figure 5:**
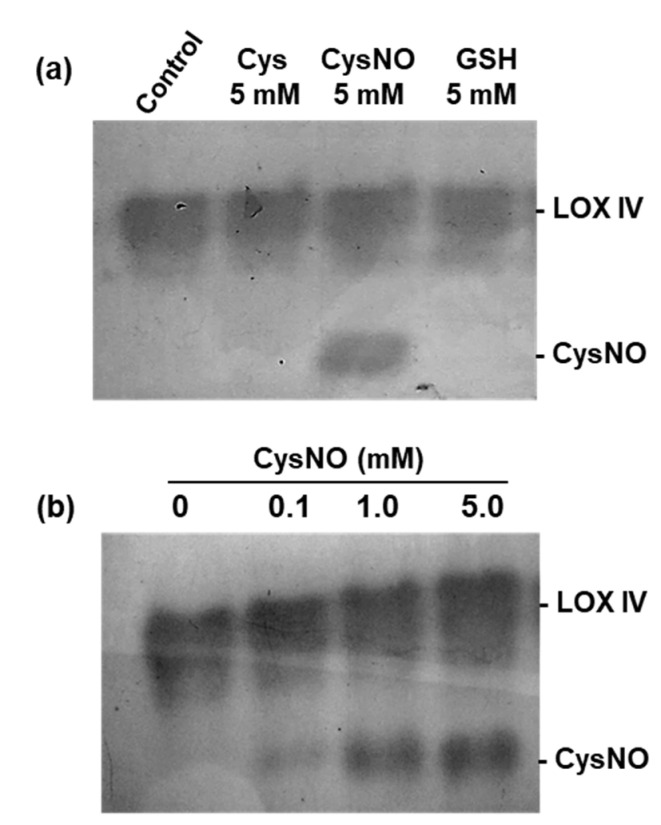
In-gel lipoxygenase activity (LOX IV) assay in sweet pepper fruits in the presence of the NO donor S-nitrosocyteine (CysSNO). (**a**) Effect of nitrosocysteine (CysSNO), cysteine (Cys) and reduced glutathione (GSH) as reductants. (**b**) Effect of CySNO concentrations on LOX IV. Treatments with Cys, CySNO and GSH were conducted by incubating the pepper fruit crude extracts with these compounds at 25 °C for 1 h before electrophoresis and further in-gel LOX activity staining. Then, protein samples were separated by native PAGE (6% acrylamide) and gels were incubated with linolenic acid and staining solution containing N,N-dimethyl-p-phenylenediamine until the appearance of pink bands (see material and methods for details).

It has been reported that, during the storage of peach fruit, NO exerted an inhibitory effect on LOX activity [26]. On the other hand, preliminary analyses on the LOX activity during pepper fruit ripening and under an enriched NO atmosphere were carried out. In that study, LOX IV increased in ripe red fruits (R) in comparison to immature green pepper (G), and NO seemed to provoke a slight increase in fruits treated with this gas [21]. Since green peppers of the sweet cultivar displayed only one single LOX isozyme, this variety was chosen to analyze the possible in vitro effect of NO and some reducing agents. Figure 5a shows that the pretreatment of crude extracts from pepper fruits with cysteine (L-Cys) increased the LOX IV activity 1.8-fold, while the NO donor nitrosocysteine (CysNO) increased the activity 3.2-fold and 4-fold with reduced glutathione (GSH) 4-fold. Pepper fruit samples were pretreated with increasing concentrations of CysNO to corroborate the potential positive effect of NO, and LOX IV activity increased by 1.9 with 0.1 mM CysNO, 2.8-fold with 1.0 mM CysNO, and 3.7-fold with 5 mM CysNO. This effect was accompanied by a change in electrophoretic mobility of LOX IV (Figure 5b). The faster band detected in the gels corresponded to the CysNO excess as it was observed when only CysNO was loaded onto the gel (results not shown).

**Figure 6:**
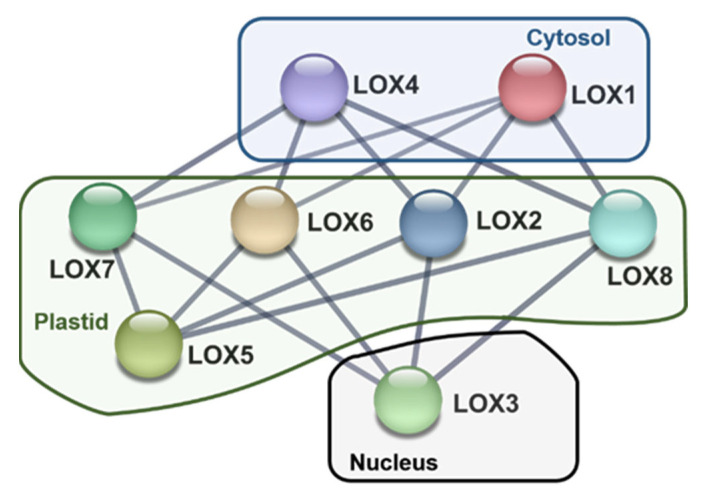
Predicted computational protein–protein interaction (PPIs) network among the eight pepper LOX isozymes encoded by the LOX genes (CaLOX1—CaLOX8) identified in the transcriptome of California-type sweet pepper fruits. The analysis was performed using STRING v11.0 with minimum required interaction score set in “medium confidence” (0.400). The proteins were assigned to either cytosol, plastids or nucleus as predicted by WoLF PSORT tools.

To better understand the relationship between the different LOXs detected in pepper and considering the eight identified genes that encode their corresponding proteins, an *in silico* analysis of their possible protein–protein interaction was accomplished considering their different subcellular locations (Figure 6). Remarkably, plastid LOXs interact with the LOXs present in cytosol and nucleus, but there is not any interaction between the LOXs present in cytosol and nucleus.

## 3. Discussion

### 3.1. LOX Isozymatic Activity Pattern Is Differently Modulated Depending on the Different Pepper Organs and the Ripening of Diverse Pepper Fruit Varieties with Different Capsaicinoid Content

LOX exerts multiple functions in higher plants and is considered a biological marker of stress because it mediates the JA biosynthesis [5,7,27,28,29]. The number of LOX isozymes and genes changes depending on the plant species, analyzed organs, and environmental conditions [30,31]. Consequently, specific analyses should be performed to identify the LOX isozyme pattern in each species and condition to evaluate their potential physiological functions. For example, during seed germination of *Vicia sativa*, two LOXs were identified and their content showed a wave during the first three days. Thus, between 0 to 25 h the two LOX activities decreased, followed by an increase until 48 h and then a decrease after 72 h. This could be related to the required FA mobilization for seedling development [32]. In soybean leaves, two LOX isozymes were detected during leaf development; however, after leaf wounding, four additional acidic LOX isozymes were identified. It was remarkable that wounding triggered an increase in the content of the four acidic LOX isozymes, both in the specific wounding locus but also in the not injured tissues. This should be correlated with their implication in the generation of JA as a signal molecule [33]. This diversity of LOX isozymes has been also described in other plant species. In chayote (*Sechium edule*) plants, five LOX isozymes were found, and their profiles were also differently modulated in roots, stems, and leaves as well as during the ripening of fruit and senescence. Thus, at fruit ripening, three LOX isozymes were recognized, but after senescence occurred a new LOX appeared [34]. In sweet pepper plant organs, a total of seven LOX isozymes were identified using C18:2 as substrate, the shoot was the organ with the higher number of isozymes, whereas green fruits only contained one unique LOX isozyme, particularly the isozyme LOX IV. This isozyme was, in turn, the most prominent in all the analyzed organs, thus suggesting its relevance in pepper plant physiology. It is remarkable that during sweet pepper fruit ripening, LOX IV activity increased, but an additional isoenzyme (LOX V) appeared. This suggests an active metabolism of FAs in the physiology of pepper fruit. Thus, it could be highlighted that the LOX activity increased during ripening, whereas an opposite behavior was observed in the content of C18:2, which was significantly diminished. A similar pattern could be also detected in the Piquillo and Alegría riojana hot pepper varieties, where the content of C18:2 also decreased during ripening while the LOX activity increased, particularly LOX IV.

The relevance of LOX activity in fruit ripening and postharvest storage has been studied in climacteric and non-climacteric fruits, where LOXs participate in aroma and flavor generation as well as in the generation of JA, a phytohormone implicated in the mechanism of wounding responses [35,36,37,38]. In the climacteric tomato (*Solanum lycopersicum*) fruits, 14 *LOX* genes have been identified [39]. Previous studies indicated that tomato *LOXA* was strongly expressed in the breaker stage, whereas the *LOXB* was highly expressed in ripe fruits. This suggests that *LOXA* contributes to changes in membrane structure that allow changes in metabolite distribution during ripening, whereas *LOXB* may be involved in the degradative process of fruit senescence [40]. On the other hand, tomato *LOXC* was expressed during ripening, which seemed to be essential for the synthesis of flavor [41], and *LOXD* was found at a very low level at the onset of ripening [37]. Furthermore, the identification of ethylene response elements in tomato gene promoter suggests that this LOX family is regulated by this phytohormone [41,42]. In apples (*Malus pumila* cv. Royal Gala), ethylene also promoted the up-regulation of the *LOX1*, *LOX3*, and *LOX7* genes, which seem to be involved the aroma production [43].

Recently, a genomic analysis based on the *Capsicum annuum* L. cv. Zunla-1 plants has allowed identifying eight *LOX* genes [44]. This corresponds to a hot pepper variety, with four of these genes (*CaLOX1*, *CaLOX3*, *CaLOX4*, and *CaLOX5*) classified as 9-LOXs, and the other ones (*CaLOX2*, *CaLOX6*, *CaLOX7*, and *CaLOX8*) as 13-LOXs [44]. LOXs are involved in the biosynthesis of oxylipins, including jasmonic acid and its derivatives, which are produced under a wide range of stress conditions, but they are also needed for plant growth and development [45,46]. Thus, the 9-LOX enzyme catalyzes the conversion of 18:2 linoleic acid/18:3 linolenic acid, to 9-hydroperoxide octadeca(di/tri)enoic acids (9-HPOD/T), respectively, while the 13-LOX enzyme catalyzes the synthesis of 13-hydroperoxide octadecatrienoic acid (13-HPOT) from linoleic/linolenic acids. Using hot pepper (*Capsicum annuum* “Nockwang”) plants, it has been shown that *CaLOX1*, which encodes a 9-specific LOX, is involved in the mechanism of defense against pathogens [47].

Capsaicinoids are a family of compounds that are exclusively produced as secondary metabolites in fruits belonging to the Capsicum genus and are responsible for the pungency of hot peppers. They are synthesized via both the phenylpropanoid and the branched-fatty-acid pathways [48,49,50,51]. To our knowledge, there is not any direct information about the correlation between LOX activity in pepper fruits and capsaicin content. In a previous study, the content of capsaicin in the pericarp and placenta of the three autochthonous Spanish varieties used in the present work was quantified at two different ripening stages [24]. Based on the total capsaicin content (µg/g fresh weight), the following ranking could be established: Piquillo with the lowest content, followed by Padrón and Alegría riojana fruits with a difference of approximately 700-fold between the highest and lowest capsaicin values in the samples assayed. Our data indicate that the number of LOX isozymes increased in hot pepper varieties, in comparison to sweet pepper. This eventuality was more outstanding in the varieties with the highest capsaicin content, Padrón and Alegría riojana. Interestingly, in animal systems, it has been reported that several products derived from the LOX activity mediated the activation of the capsaicin-activated channel of sensory neurons that cause severe pain [4], a field that years later allowed Professor David Julius to be awarded the Nobel Prize 2021 in Physiology and Medicine. Accordingly, further research to elucidate the intimate mechanisms by which LOXs and capsaicinoids interact will provide interesting data, not only for horticultural biotechnology but also for medicine.

### 3.2. NO Gas Differentially Modulates LOX Genes and LOX4 Activity during Ripening

The available information on NO on the LOX systems is very scarce. In an early proteomic study of potential targets of *S*-nitrosation in potato tuber samples treated with the NO donor *S*-nitrosoglutathione (GSNO), it was possible to identify 80 protein candidates and, among them, was found a LOX protein [52]. The NO treatment of the cut *Consolida ajacis* flower during postharvest storage triggered a decrease in LOX activity, which alleviated its deterioration since the NO promoted the membrane stability [53]. In Arabidopsis, NO induces the expression of the *LOX3* gene, which leads to JA production [54]. However, the effect of exogenous NO application on the fruit LOX activity and gene expression is still very limited. Recently, in tomato fruits exposed to NO during ripening, an increase was found in the expression of genes encoding LOXC, hydroperoxide lyase (HPL), and alcohol dehydrogenase (ADH2) enzymes, all three related to the biosynthesis of FA volatiles associated with tomato flavor [25]. In peach fruits, during cold storage, exogenous NO inhibited the LOX activity, and the expression of three *LOX* genes (*LOX1*, *LOX2*, and *LOX3*) were first up-regulated and then down-regulated after a week of NO treatment. This effect was the opposite in *LOX4*, which was first down-regulated and then up-regulated [55]. In our experimental model of pepper fruit ripening under a NO gas atmosphere, it was found that NO affected differentially the expression of the eight *LOX* genes detected in the fruit but, interestingly, this gas only had a slightly positive effect on LOX IV activity [21]. However, using an *in vitro* approach, positive modulation of NO on the LOX IV activity was corroborated. This indicates that LOX IV is susceptible to undergoing *S*-nitrosation, a post-translational modification (PTM) promoted by NO that is common during pepper fruit ripening [56]. Considering that sweet pepper fruit mainly contains LOX IV, it could be considered the outstanding relevance of this isozyme to be correlated with the synthesis of jasmonic acid through the metabolism of PUFA during the fruit ripening process. This, therefore, places LOX IV in the crossroad of the regulatory roles attributed to this phytohormone. In this context, very recently it has been described that passion fruit (*Passiflora edulis*) has a total of 12 *PeLOX* genes whose expression change in the different organs and under several stress conditions [38]. However, it was remarkable that the *PeLOX4* expression, as well as the total LOX activity in those fruits, increased significantly during the ripening process suggesting that this could be the candidate gene in the formation of volatile compounds that contribute to their flavor [38].

It should be pointed out that, despite detecting eight *LOX* genes in sweet pepper fruits, only a single band of LOX activity was observed, which suggests that it is always necessary to complement molecular and biochemical data to obtain a more complete understanding of the involvement of this enzymatic system in the fruit physiology.

## 4. Materials and Methods

### 4.1. Plant Material and Growth Conditions

California-type sweet pepper (*Capsicum annuum* L., cv. Melchor) fruits were obtained from Zeraim Iberica/Syngenta Seeds, Ltd. experimental greenhouses (Roquetas de Mar/El Ejido, Almería, Spain). Additionally, fruits, at two different ripening stages, of three autochthonous Spanish varieties that have different pungency levels, including Padrón, Piquillo, and Alegría riojana, were also supplied by their corresponding Regulatory Councils of Denomination of Origin “Pemento de Herbón” (La Coruña, Spain) and Piquillo (Navarra, Spain) [24]. In all cases, the fruits used were randomly collected and without any visible damage. In the laboratory, fruits were washed with distilled water, dried with filter paper, and cut into vertical strips (using only the pericarp), immediately frozen with liquid nitrogen, and then stored at −80 °C.

In the analyses of the different plant organs (roots, stems, and leaves), seeds of sweet pepper, cv. Melchor, obtained from Syngenta Seeds Ltd. (El Ejido, Almería, Spain) were germinated in Petri dishes containing Murashige and Skoog medium for 5 d at 30 °C in the dark. Then, the healthiest seedlings having similar sizes and appearance were transferred to hydroponic culture (four seedlings in 1 L container) and grown for an additional 25 days at 22/18 °C during 16 h photoperiod and irradiance of 100–120 µmol m^−2^ s^−1^ [57,58].

For assays of exogenous NO gas treatment, California-type sweet pepper fruits were collected from plants grown in plastic-covered greenhouses. Fruits without any external damages were selected at three developmental stages: green immature (G), breaking point (BP1), and red ripe (R). For the application of NO, two additional groups were set: fruits treated with 5 ppm NO for 1 h (BP2 + NO) and another group that was not treated with NO (BP2 − NO), which was used as control of BP2 + NO [21,23,59]. After 3 days, all fruits were chopped into small cubes (5 mm/edge), frozen under liquid nitrogen, and stored at −80 °C until use. Supplementary Figure S1 shows a representative picture of the experimental design followed in this study with the representative phenotypes of sweet pepper fruits at different ripening stages and subjected to NO treatment [23].

### 4.2. Preparation of Crude Extracts

The pepper plant organs were ground in liquid N_2_ using a mortar and pestle, and the resulting powder was suspended in 0.1 M Tris-HCl buffer, pH 8.0, containing 1 mM EDTA, 0.1% (*v*/*v*) Triton X-100, 10% (*v*/*v*) glycerol to a final plant material/buffer (*w*/*v*) ratio of 1:1. Homogenates were then filtered through two layers of Miracloth and centrifuged at 27,000 g for 20 min. The supernatants were used for enzymatic assays.

### 4.3. Gas Chromatography-Mass Spectrometry (GS-MS) Assay of Fatty Acids (FAMEs)

Total lipids were extracted in triplicate based on a previous method [21] using the pericarp of the fruits. Briefly, 2.5 g of pepper fruit samples (previously frozen and ground until obtained a fine powder) were re-suspended into 25 mL of chloroform and methanol (2:1, v:v) solution with continuous shaking for 1 h. Then, the solution was centrifuged at 5000 g for 10 min at 25 °C, and the supernatant was subjected to the same process, at least twice, until it became colorless. After that, 15 mL 0.85% (*w*/*v*) NaCl was added to supernatants and mixed gently in a decanting ampoule. The lower organic phase was collected into a pre-weighted glass tube, evaporated to dryness under an N_2_ flow and the total lipid content was then determined gravimetrically. Subsequently, the samples were derivatized using 1 mL boron trifluoride-MeOH for 15 min at 60 °C to obtain the corresponding fatty acid methyl esters (FAMEs). After cooling at room temperature, 700 μL hexane and 700 μL Milli-Q water was added and mixed strongly for 5 min. Then, 500 μL of the organic phase was transferred to a chromatography vial for loading on a Varian 450 GC 240 MS system such as was previously described by González-Gordo et al., 2019 [21]. The mass spectrometer worked in electron ionization mode and mass spectra were acquired between 50 and 1000 arbitrary units of mass. Fatty acids were identified based on the similarity with the NIST08 mass spectral library and using standards injected in the same conditions.

### 4.4. In-Gel LOX Activity Assay and Isozyme Profile

In-gel assay of lipoxygenase (LOX) activity was determined according to Heinish et al. (1996) [60] with minor modifications [21]. Briefly, pepper samples were separated using non-denaturing polyacrylamide gel electrophoresis (PAGE) on 6% acrylamide gels. After electrophoresis, gels were briefly rinsed in distilled water, and incubated at 4 °C for 1 h under continuous shaking, in darkness, with a solution, containing 0.2 M glycine-sodium hydroxide buffer (pH 9.0) and 50 μL of linolenic acid (C18:2, Sigma) prepared in 50 μL of ethanol. Subsequently, the gel was again rinsed briefly with distilled water and incubated with 20 mL staining solution containing 0.2 g N,N-dimethyl-p-phenylenediamine, 1.8 mL methanol, and 0.2 mL acetic acid. Gels were shaken slightly at room temperature until the appearance of pink bands representing enzyme activity. Bands were quantified by using ImageJ software.

### 4.5. RNA Isolation and Semiquantitative RT–PCR

Total RNA was extracted with Trizol according to instructions provided by Gibco BRL (Life Technologies); 5 μg of total RNA was used to produce cDNA for the reverse transcriptase (RT) reaction by adding 0.5 mM dNTPs, poly-dT23, 5x RT buffer, 40 U RNase inhibitor (Invitrogen) and 200 U Reverse Transcriptase (Thermo Fisher) in a final volume of 20 μL. The reaction was carried out at 50 °C for 30 min. Actin (*CaACT*) and glyceraldehyde-3-phosphate dehydrogenase (*CaGAPDH*) from pepper were used as housekeeping genes for semiquantitative RT–PCR [22]. Table 1 shows the oligonucleotides used for the amplification analyses of the eight pepper *CaLOX* genes identified on the sweet pepper transcriptome previously reported [21]. PCR products were then detected after electrophoresis in 2.8% (*w*/*v*) agarose gels and by staining with GelRed™. Quantification of the bands was performed using a Gel Doc system (Bio-Rad Laboratories) coupled with a high-sensitivity camera.

### 4.6. In Vitro Treatment with Nitric Oxide (NO) Donors, and Reducing Agents

For the *in vitro* assays, before non-denaturing PAGE, red pepper fruit samples were incubated at 25 °C for 1 h with different concentrations of diverse potential enzyme activity modulators including *S*-nitrosocysteine (CysNO); L-cysteine (L-Cys) and reduced glutathione (GSH). In all cases, the solutions were made up fresh before use.

### 4.7. Analysis of the Protein–Protein Interaction (PPI) Network and Subcellular Localization

The protein localization based on their amino acid sequences was predicted using WoLF PSORT (https://wolfpsort.hgc.jp/ (accessed on 30 October 2022)) [61,62]. LOX proteins were used as inputs for PPI network and pathway enrichment analysis. The STRING database, version 11.0 (https://string-db.org/ (accessed on 30 October 2022)) was used to assess the protein functional association [63], and a confidence view was generated by setting the filter to medium confidence (0.400).

### 4.8. Statistical Analyses and Other Assays

Data are presented as the mean ± SEM of at least three independent biological replicates. Pairwise analysis of variance (ANOVA) was used to detect differences between samples with the aid of the Statgraphics Centurion program.

Protein concentration was determined using the Bio-Rad protein assay (Hercules, CA, USA), with bovine serum albumin as standard. Bands’ intensities were quantified using ImageJ 1.45 software.

## 5. Conclusions

Lipoxygenases encompass a very diverse family involved in a multitude of functions from germination and development, as well as in response mechanisms to environmental stresses [27,28,64,65,66,67,68]. Pepper lipoxygenases show a complex regulation that depends on the pepper plant organ. In fruits, LOX regulation is associated with ripening [69] and it could contribute to some organoleptic properties such as aroma [38,70]. In pepper, the LOX regulation seems to take place at the transcriptional, translational, and post-translational levels. Remarkably, the LOX isozymatic activity pattern changes differentially in the ripening of sweet and hot peppers where the number of LOX isozymes increases with the content of capsaicin, and this opens new questions about the potential correlations between LOX and capsaicin biosynthesis, a field that may have interdisciplinary approaches, including biotechnology, pharmacology, and medicine. Furthermore, exogenous NO gas promotes a slight increase of LOX activity, but under *in vitro* assays, using the NO donor *S*-nitrosocyteine, it was found that *S*-nitrosation of LOX IV may be a relevant event that could be involved in the fruit physiology through the roles attributed to jasmonic acid.

## Figures and Tables

**Table 1:** Oligonucleotides used for the semiquantitative RT–PCR analysis of *CaLOX* genes. Letters “F” and “R” correspond to forward and reverse oligonucleotides, respectively.

Gene	Oligonucleotides (5′ to 3′)	Product Size (bp)	NCBI ID	Uniprot ID
*CaLOX1*	F: TTATGCCAGAGCCAGGAACT	182	LOC107847934	-
	R: CTTTGTCCATTCAGGCGATT			
*CaLOX2*	F: GCCATTTCTGGATCGGATTA	171	LOC107861665	A0A1U8FUC8
	R: GCATCAACAGGTGGTGTGAC			
*CaLOX3*	F: GTATCCCGCACATCGCTACA	141	LOC107864114	A0A1U8G3E4
	R: TTGAGCATGCCAGAACCTGT			
*CaLOX4*	F: GCCAAGTCCACCACAGCTAA	189	LOC107865522	A0A1U8GGP5
	R: CGTCCGTCAAGTCCAAGACA			
*CaLOX5*	F: TGAACAAGGTGTCCGCCTAC	156	LOC107879335	A0A1U8HER1
	R: CACCAGGCTTGGAGTTCAGT			
*CaLOX6*	F: TGGAGCTGGTATTGTGCCTT	139	LOC107874182	A0A1U8GZH6
	R: GGGCTTGTATCATACTTCATGT			
*CaLOX7*	F: TCACCACAATCTACAAAACCCT	163	LOC107874197	A0A1U8GZ84
	R: CAGCCTTAGTGCTATTTGCAGC			
*CaLOX8*	F: AAGTGGTGCTGGTGTTCCTC	176	LOC107847668	A0A1U8EVZ2
	R: TTGCATGCTGCCAAGTTCCA			
*CaACTIN*	F:CAAACAGGTTTTAAAAGATGGCAGATGAAG	172	LOC107840006	-
	R:TCCTTTTGACCCATCCCTACCATAACAC			
*CaGAPDH*	F: CGACAACGAGTGGGGTTACA	113	LOC107845282	-
	R:CTTGCGCCAACTTCTGCATT			

## Data Availability

Sequence Read Archive (SRA) data are available at the following link https://www.ncbi.nlm.nih.gov/sra/PRJNA668052 (accessed on 28 May 2020).

## Supplementary Materials

The following supporting information can be downloaded at: https://www.mdpi.com/article/10.3390/ijms232315211/s1.
