# Supplementary material for: Lipoxygenase (LOX) in Sweet and Hot Pepper (Capsicum annuum L.) Fruits during Ripening and under an Enriched Nitric Oxide (NO) Gas Atmosphere
Source: Int J Mol Sci. 2022 Dec 2;23(23):15211. doi: 10.3390/ijms232315211 (PMC9740671; doi:10.3390/ijms232315211)
Supplement: Supplementary file 1 [file ijms-23-15211-s001.zip › ijms-2035609-supplementary.pdf]

International Journal of
Molecular Sciences

MDPI

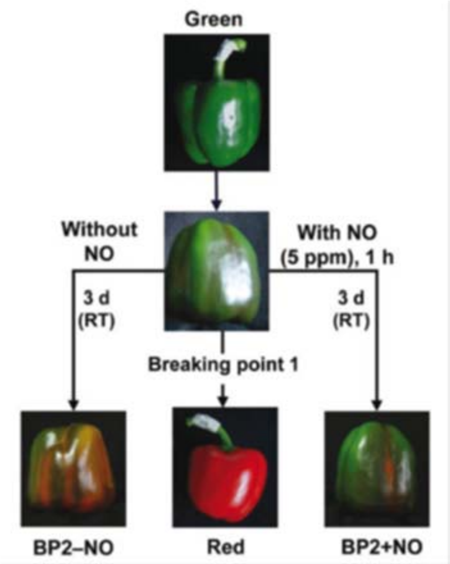

Supplementary Figure S1. Illustrative picture showing the experimental design used in this study with the representative phenotype of sweet pepper (*Capsicum annuum* L.) fruits at different stages and treatments: immature green, breaking point 1 (BP1), breaking point 2 without NO treatment (BP2 – NO), breaking point 2 with NO treatment (BP2 + NO), and ripe red. Fruits were subjected to a NO-enriched atmosphere (5 ppm) in a hermetic box for 1 h and were then stored at room temperature (RT) for 3 days. Reproduced with permission from González-Gordo et al. (2022).
